# Design of a Multi-Sensor System for Exploring the Relation between Finger Spasticity and Voluntary Movement in Patients with Stroke

**DOI:** 10.3390/s22197212

**Published:** 2022-09-23

**Authors:** Bor-Shing Lin, I-Jung Lee, Pei-Chi Hsiao, Shu-Yu Yang, Chen-Yu Chen, Si-Huei Lee, Yu-Fang Huang, Mao-Hsu Yen, Yu Hen Hu

**Affiliations:** 1 Department of Computer Science and Information Engineering, National Taipei University, New Taipei City 237303, Taiwan; 2 College of Electrical Engineering and Computer Science, National Taipei University, New Taipei City 237303, Taiwan; 3 Department of Physical Medicine and Rehabilitation, Chi-Mei Medical Center, Tainan 71004, Taiwan; 4 Department of Physical Medicine and Rehabilitation, Taipei Veterans General Hospital, Taipei 112, Taiwan; 5 Faculty of Medicine, National Yang Ming Chiao Tung University, Taipei 112, Taiwan; 6 Department of Computer Science and Engineering, National Taiwan Ocean University, Keelung City 202301, Taiwan; 7 Department of Electrical and Computer Engineering, University of Wisconsin-Madison, Madison, WI 53706, USA

**Keywords:** stroke, voluntary movement, spasticity, inertial measurement unit, wearable, modified Ashworth scale

## Abstract

A novel wearable multi-sensor data glove system is developed to explore the relation between finger spasticity and voluntary movement in patients with stroke. Many stroke patients suffer from finger spasticity, which is detrimental to their manual dexterity. Diagnosing and assessing the degrees of spasticity require neurological testing performed by trained professionals to estimate finger spasticity scores via the modified Ashworth scale (MAS). The proposed system offers an objective, quantitative solution to assess the finger spasticity of patients with stroke and complements the manual neurological test. In this work, the hardware and software components of this system are described. By requiring patients to perform five designated tasks, biomechanical measurements including linear and angular speed, acceleration, and pressure at every finger joint and upper limb are recorded, making up more than 1000 features for each task. We conducted a preliminary clinical test with 14 subjects using this system. Statistical analysis is performed on the acquired measurements to identify a small subset of features that are most likely to discriminate a healthy patient from patients suffering from finger spasticity. This encouraging result validates the feasibility of this proposed system to quantitatively and objectively assess finger spasticity.

## 1. Introduction

Finger spasticity is a common motor disorder, which causes involuntary muscle contraction and severely affects patients with stroke in their daily activities [1,2]. Research has shown that quantifying finger spasticity is critical for the early intervention, rehabilitation, and treatment of patients with stroke [3].

The modified Ashworth scale (MAS) [4] is typically used for assessing the severity of spasticity in medical institutions due to its convenience. The physician stretches part of the participant’s body and assesses the resistance during passive stretching to obtain measurements of MAS [5]. Although the MAS is convenient to apply, it is subjective and has poor interrater reliability [6], that is, the results from one evaluator may differ from those of other evaluators. Although the interrater reliability of MAS has been reported in the previous studies, it is still the most accepted clinical tool to measure the increase in muscle tone [7]. Moreover, the conventional MAS assessment focused on the measurement of muscle tone during passive stretching but did not investigate the relations between spasticity and voluntary movements, which is critical for investigating how spasticity affects the participants’ daily movements. Therefore, the development of an objective spasticity assessment system is necessary for assisting physicians in assessing the spasticity severity of fingers during voluntary movements.

To date, several automatic systems using various sensors, such as inertial measurement units (IMUs) and surface electromyography (sEMG) systems, have been proposed for assisting the assessment of the upper-limb spasticity. In 2012, Park et al. [8] developed a machine that could record position, velocity, and torque data during quick passive stretching. Four tactile models were then developed to standardize passive stretching and enhance interrater reliability. In 2018, Ang et al. [9] proposed an objective evaluation system for the severity of spasticity. Three IMUs on the upper limb were used to collect information of joint angle, velocity, and acceleration from the upper-limb motion; these data were applied to an upper-limb model to predict a velocity-dependent tonic stretch reflex threshold. The results revealed that the predicted threshold was strongly correlated with the MAS and could be regarded as an indicator of spasticity. In 2019, Park et al. [10] proposed a system that uses a multilayer perceptron to predict MAS scores. Manual spasticity evaluation was performed to record the joint angle and force when a patient’s forearm was passively stretched. Nine features were extracted from the angle and force information, and the extracted features were input to a multilayer perceptron to predict the patient’s MAS score. The critical features for evaluating the MAS were also determined in the studies of Park et al. Zhang et al. [11] used IMUs and sEMG to collect data on patients with various MAS scores during an assessment through quick passive stretching. The system combined single-variable and multivariable linear regression with support vector regression to predict MAS scores. In 2020, Kim et al. [12] used IMUs to collect acceleration and rotation information of patients’ elbows during stretching by physicians, and the collected information was input into five common machine learning models for automatic MAS score prediction. The results of the five models were compared to determine which model performed the best. These studies have all focused on using the kinematic and force information during passive forearm stretching to assess the severity of upper-limb spasticity but did not assess spasticity of fingers. Moreover, the aforementioned studies assessed the upper-limb spasticity by collecting the sensor data when the therapist performed quick passive stretching on the patients, which could not reveal how the spasticity affects the voluntary movements.

Therefore, to research how upper-limb spasticity affects the patient’s voluntary movement, few studies have proposed wearable systems to assess voluntary movements by patients with stroke. Koh et al. [13] presented an approach based on stepwise regression to investigate the recovery progress of voluntary movement. Bai et al. [14] proposed a system of assessing the upper-limb voluntary motion before and after botulinum toxin treatment, which is a common treatment for spasticity. Chen et al. [15] used data on voluntary movements to assess spasticity at the elbow. IMUs and sEMG were used to collect information about the movements while participants performed repetitive tasks. The collected information was input to the random forest algorithm to predict MAS scores. The predicted MAS scores were compared with the physician-assessed MAS scores and had an accuracy of 0.979. However, although the aforementioned system assessed the spasticity with voluntary movements, they only assessed the spasticity of wide joints of upper-limb but did not assess the spasticity of fingers, which also greatly impedes the patient’s manual dexterity when they perform delicate tasks with their fingers. Furthermore, the wearable devices adopted in the previous research were not suitable to be applied to detect the movement of fingers. Therefore, a new wearable device for detecting the hand movement is needed.

To investigate the difference of the upper-limb motion among different levels of finger spasticity, this study proposes a wearable system that combines multiple sensors, including IMUs and a pressure ball, to capture the motion and force data for the subject’s upper-limb voluntary movement. Seven patients with stroke with low-to-moderate spasticity and seven healthy participants were asked to wear the wearable system and voluntarily perform five tasks, and several features based on descriptive statistics were extracted from the collected sensor data. The significant features for differentiating patients with different spasticity scores were investigated with statistical approach.

## 2. Materials and Methods

### 2.1. Hardware Design

**Figure 1:**
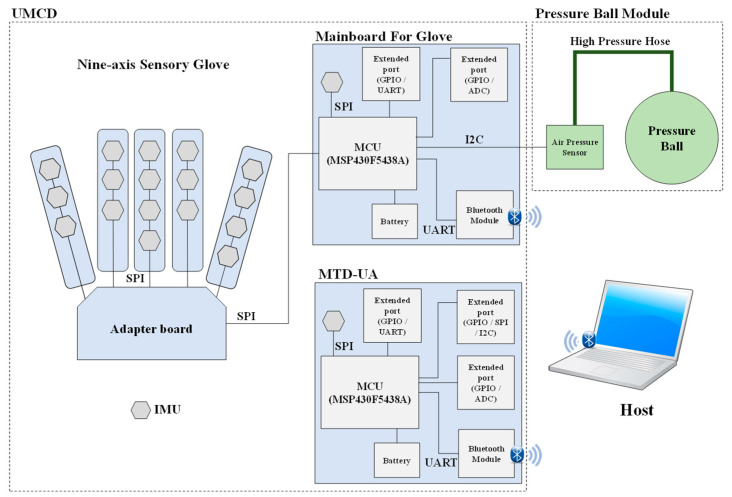
System architecture.

**Figure 2:**
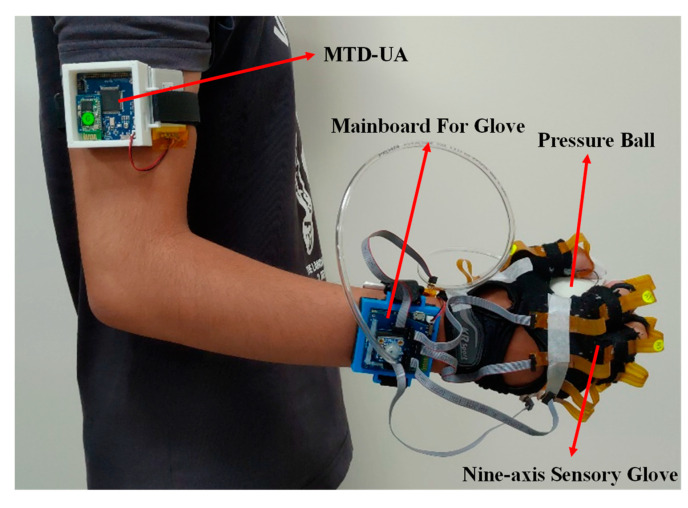
Prototype of the proposed system.

The architecture of the proposed system is presented in Figure 1. A prototype of the proposed system is displayed in Figure 2. The system includes an upper limb motion capture device (UMCD) and a pressure ball module. The UMCD comprises a nine-axis sensory glove and a motion tracking device for the upper arm (MTD-UA). The nine-axis sensory glove contains 18 IMUs (MPU9250, InvenSense, San Jose, CA, USA) for capturing hand motion. The sensory glove combined with the pressure ball is used to detect differences in air pressure in a soft tennis ball caused by a participant gripping the ball. In the MTD-UA, an IMU is used to capture the motion of the upper arm. The microcontrollers (MCUs; MSP430F5438A, Texas Instruments, Dallas, TX, USA) on the mainboard of the sensory glove and the MCU of the MTD-UA collect information from the aforementioned sensors and encapsulate the data into a packet. Packets are sent to the host program through Bluetooth (HL-MD08R-C2-AT, Hotlife, Taipei, Taiwan) at a sampling frequency of 50 Hz. The data from the packet are extracted at the host using a software program and then stored.

#### 2.1.1. UMCD

**Figure 3:**
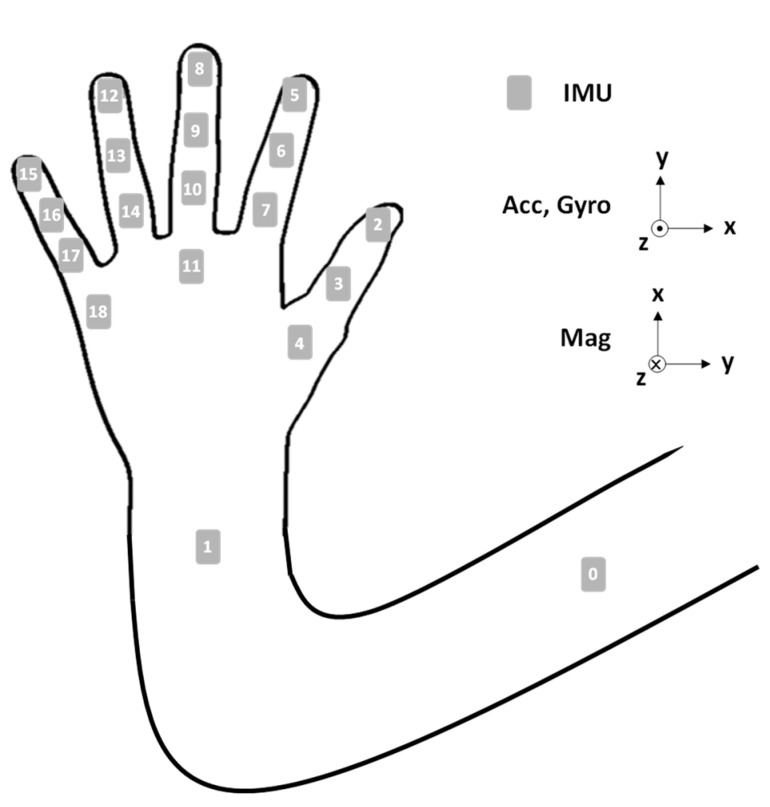
Sensor positions and ROMs.

The UMCD comprises the nine-axis sensory glove and an MTD-UA. Eighteen IMUs are fixed on the sensory glove to detect hand motions, and one IMU is placed on the MTD-UA to capture the motion of the upper arm. The positions of the 19 IMUs are displayed in Figure 3.

**Figure 4:**
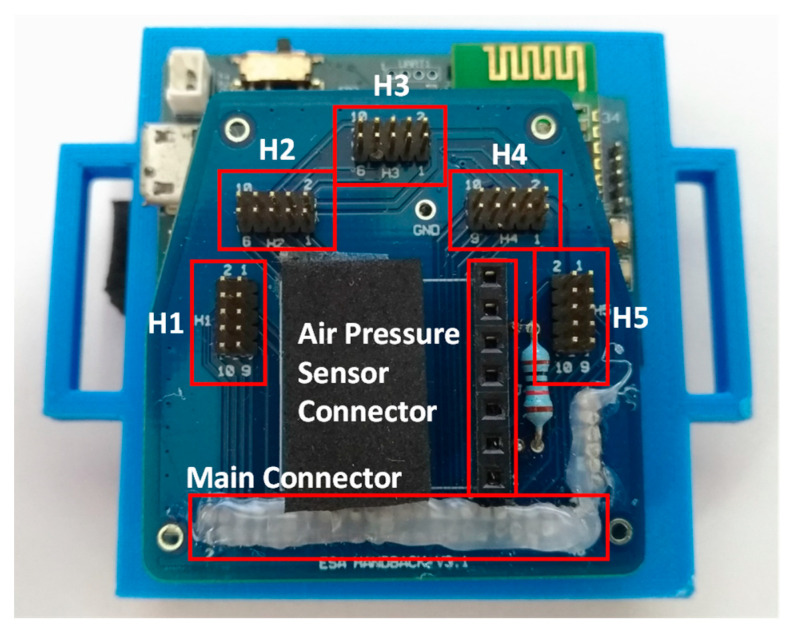
Combination of the modified adapter board and mainboard of the sensory glove.

The design of the nine-axis sensory glove was introduced in [16]. Each IMU provides three-axis acceleration, angular velocity, and magnetic field data. The adaptor board for the sensory glove was modified and is combined with the pressure ball module (Figure 4). H1–H5 have the same functions as those in [16]; they can communicate with the five flexible IMU boards. The air pressure connector is used to communicate with the air pressure sensor through the inter-integrated circuit (I2C) interface. The purpose of the main connector is to combine the data from the five flexible IMU boards and air pressure sensor and to send the combined data to the MCU.

The MTD-UA is a mainboard without the sensory glove module. The purpose of the MTD-UA is to capture the motion of the upper arm while the participant is performing a task. The data from the upper arm can be combined with the data from the IMU on the mainboard of the sensory glove to calculate the angle of the elbow. By combining the data from the sensory glove and MTD-UA, the system can capture the elbow, wrist, and hand motions simultaneously.

#### 2.1.2. Pressure Ball Module

**Figure 5:**
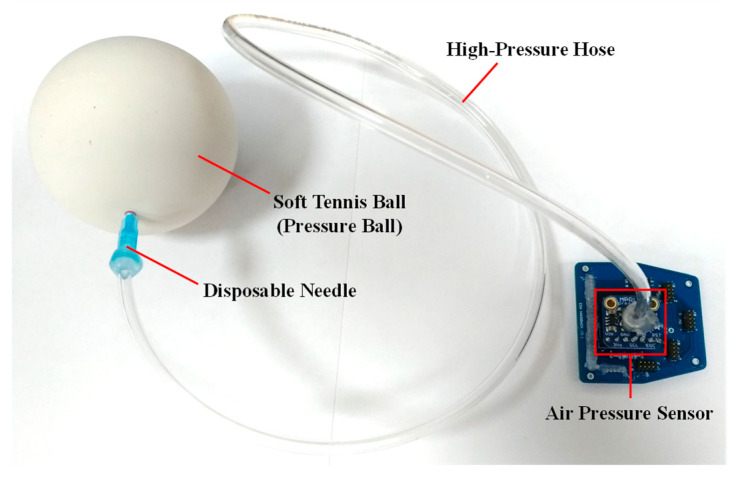
Pressure ball module and adapter board.

The pressure ball module comprises four parts, namely an air pressure sensor (MPRLS Ported Pressure Sensor Breakout, Adafruit Industries, New York City, NY, USA) [17], a high-pressure hose, a disposable needle, and a soft tennis ball. The air pressure sensor is connected to an adapter board to communicate with the MCU on the mainboard of the sensory glove. A photograph of the pressure ball module is presented in Figure 5. The purpose of the pressure ball module is to detect air pressure in the soft tennis ball when it is squeezed by the participant. When the participant applies force to the ball, the air in the ball reaches the air pressure sensor though the high-pressure hose. The air pressure sensor detects this air pressure and sends the detected value to the MCU on the mainboard through the I2C. The MCU then combines the air pressure value with the data from the sensory glove into a packet and sends the packet to the host program through Bluetooth.

Because atmospheric pressure (AP) varies between times and locations, the initial air pressure and hardness of the ball vary; therefore, calibration of the ball is necessary. Before the participant performs the task, a disposable needle (Figure 5) is removed from the ball and exposed to the air to detect the current AP; this value is used as a reference. The needle is then reinserted into the pressure ball, and the difference between the AP and the pressure in the pressure ball is calculated according to the following formula:

(1) $$p^{\prime }=p-p_{a}$$

Here, *p* is the air pressure value in the air pressure sensor, *p*_a_ is the current AP, and *p*′ is the difference between *p* and *p*_a_. To ensure that the ball has similar hardness in every environment, a threshold value of *p*′ (±0.5% *p*_a_) was adopted. If *p*′ is less than −0.5% *p*_a_, the pressure ball is too soft, and more air is required; if *p*′ is greater than 0.5% *p*_a_, the pressure ball is too hard, and some air must be released from the pressure ball. Through the use of this calibration process, the hardness of the pressure ball can be adjusted in accordance with the current AP.

### 2.2. Software Design

The program is developed in C# programming language and runs on the host laptop. After receiving the packets from the UMTS, the program immediately parses the data packet into the values of the acceleration, angular velocity, magnetic field, and air pressure. After parsing the data packet and obtaining the data from all the sensors, the calibration of each sensor is performed to ensure the reliability of the sensor data. Additionally, the calibrated sensor data are then processed using the sensor fusion algorithm to estimate the quaternion of attitude at the current time. The Euler angle of each IMU sensor and the joint angle between every two adjacent IMU sensors can be calculated from the quaternion of attitude. Furthermore, the calibrated air pressure is simultaneously calculated from the data from the air pressure sensor.

**Figure 6:**
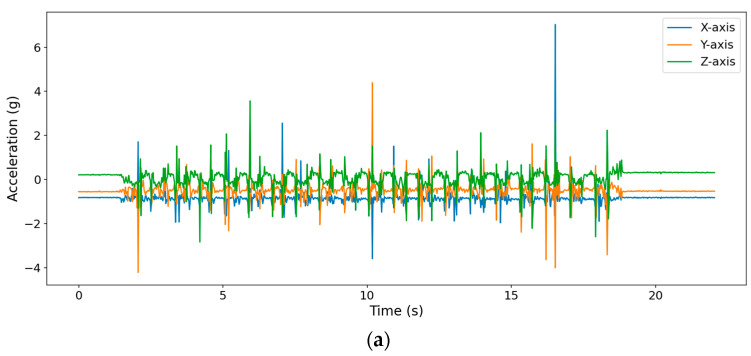

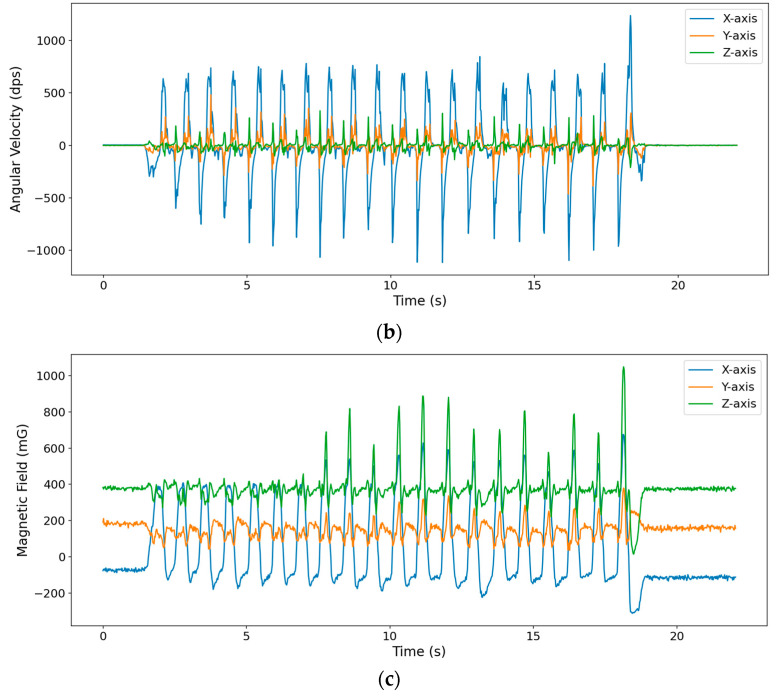
The nine-axis raw data collected from IMU8: (**a**) three-axis acceleration, (**b**) three-axis angular velocity, and (**c**) three-axis magnetic field.

Figure 6 demonstrates nine-axis raw data collected from the IMU on the distal segment of middle finger (IMU8) while performing 20 repetitions of ball squeezing. The nine-axis data includes three-axis acceleration, three-axis angular velocity, and three-axis magnetic field, which are presented in Figure 6a–c. It can be clearly noticed that there are 20 peaks and valleys in angular velocity magnetic field on *x*-axis because the finger flexes and extends mainly around the *x*-axis of the IMU.

#### 2.2.1. Sensor Calibration

Because the noise exists in all the IMUs, to obtain more reliable values from the IMUs, all the IMUs had to be calibrated before being used. The calibration process of the accelerometer and gyroscope is automatically conducted when the sensor glove is switched on and lay on the desk. Since the magnetometers are easily affected by the magnetic disturbance in the environment, it is important to calibrate the magnetometer before using the value from the magnetometer data. To reduce the complexity of computation when calibrating magnetometer, an effective calibration method was adopted [18]. The calibration process of magnetometer is performed on the program. The sensor glove and MTD-UA were rotated in 8-shape to collect the first 2000 samples of magnetic field data for each axis when the devices started to transmit data to the host program. After obtaining the 2000 samples of magnetic field, two steps of calibration, including hard-iron and soft-iron calibrations, were performed. Hard-iron calibration eliminates the distortion due to the constant magnetic field caused by the earth’s magnetic field. Soft-iron calibration eliminates the distortion due to the existing magnetic field. The formulas to obtain the offsets for hard-iron and soft-iron calibration are expressed in (2) to (5). *S_x_*, *S_y_*, and *S_z_* denote the 2000 magnetic field data series along three axes, respectively. αx, αy, and αz denote the bias of hard-iron distortion along three axes, respectively. *β_x_*, *β_y_*, and *β_z_* denote the scale factor to rescale the magnetic data along three axes, respectively. *σ_x_, σ_y_,* and *σ_z_* denote the max chord of length of the three axes, respectively. After obtaining the bias and scale factor, the calibrated magnetic field data can be calculated by (5). $m_{y}^{\prime }$, and $m_{z}^{\prime }$ represented the calibrated magnetic field data.

(2) $$\left[\begin{array}{c} \alpha_{x}\\ \alpha_{y}\\ \alpha_{z} \end{array}\right]=\frac{1}{2}\cdot \left[\begin{array}{c} \max S_{x}+\min S_{x}\\ \max S_{y}+\min S_{y}\\ \max S_{z}+\min S_{z} \end{array}\right]$$

(3) $$\left[\begin{array}{c} \sigma_{x}\\ \sigma_{y}\\ \sigma_{z} \end{array}\right]=\frac{1}{2}\cdot \left[\begin{array}{c} \max S_{x}-\min S_{x}\\ \max S_{y}-\min S_{y}\\ \max S_{z}-\min S_{z} \end{array}\right]$$

(4) $$\left[\begin{array}{c} \beta_{x}\\ \beta_{y}\\ \beta_{z} \end{array}\right]=\frac{\sigma_{x}+\sigma_{y}+\sigma_{z}}{3}\cdot \left[\begin{array}{c} \frac{1}{\sigma_{x}}\\ \frac{1}{\sigma_{y}}\\ \frac{1}{\sigma_{z}} \end{array}\right]$$

(5) $$\left[\begin{array}{c} m_{x}^{\prime }\\ m_{y}^{\prime }\\ m_{z}^{\prime } \end{array}\right]=\left[\begin{array}{c} \beta_{x}\left(m_{x}-\alpha_{x}\right)\\ \beta_{y}\left(m_{y}-\alpha_{y}\right)\\ \beta_{z}\left(m_{z}-\alpha_{z}\right) \end{array}\right]$$

#### 2.2.2. User Interface for Data Collection

After connecting with and receiving data from the UMTS, the software showed the IMU data, estimated joint angle and air pressure in real-time on the GUI. A drop-down menu let users select the IMU data they would like to observe. For example, if IMU 8 is selected, the nine-axis data and current attitude of IMU8 will be presented on the GUI. All the estimated joint angles are also shown on the GUI for clinicians to observe the subject’s movement of the specific finger segment. In addition to the data of IMUs, the current air pressure detected by the air pressure module is also shown on the GUI.

## 3. Clinical Experiments and Data Analysis

Seven healthy participants (N1–N7) and seven patients with stroke (S1–S7) were recruited in this study. All experiments were performed in the Chi-Mei Medical Center, Tainan, Taiwan. All procedures and measurements were performed in accordance with the World Medical Association’s Declaration of Helsinki: Ethical Principles for Medical Research Involving Human Subjects (version October 2013) and approved by the Institutional Review Board (IRB) of Chi-Mei Medical Center, Tainan, Taiwan (IRB code: 11002-007). Prospective participants were included if they (1) were aged 20–80 years and (2) could sit on a chair for at least 40 min. A prospective participant was excluded if they (1) had symptoms of unilateral neglect or attention deficit, (2) had cognitive or language impairments and could not understand and perform the specified tasks, (3) had upper-limb impairment caused by bone joints or peripheral nervous system lesions prior to onset of stroke, or (4) had been diagnosed with dementia or depression. After the participant signed the informed consent, one therapist tested the spasticity of metacarpophalangeal (MCP) joints for the participant’s index, middle, ring, and little fingers simultaneously on the affected side according to MAS. The demographic characteristics of the participants are listed in Table 1. The mean and standard deviation of the age of the healthy participants were 38.78 ± 18.64 years old, whereas those of the patients with stroke were 60.00 ± 13.36 years old. All participants were right-handed. The right sides of four of the patients with prior stroke were affected; the left side was affected in the other three patients.

After the MAS scores of fingers were obtained, the researcher assisted the participant in putting on the UMCD and ensured that the IMUs were in the correct position on the finger segments and upper arm without misalignment. After the participants put on the UMCD, the researchers then asked the participant to perform five tasks: cone stacking (CS), slow flexion and extension (S-FE), fast flexion and extension (F-FE), slow ball squeezing (S-BS), and fast ball squeezing (F-BS). The five tasks were selected because they are the most common tasks for occupational therapy, which means that most stroke patients are familiar with the specified tasks and could understand how to perform the tasks more easily. The patients with stroke performed the five tasks with their affected side, and the healthy participants performed the five tasks with their dominant side. Before each task, the participant placed the test hand on a table of height 75–80 cm. The height of the table was adjusted according to the participant’s height, and the legs of the table were fastened to the floor to prevent unexpected movement.

### 3.1. CS Task

**Figure 7:**
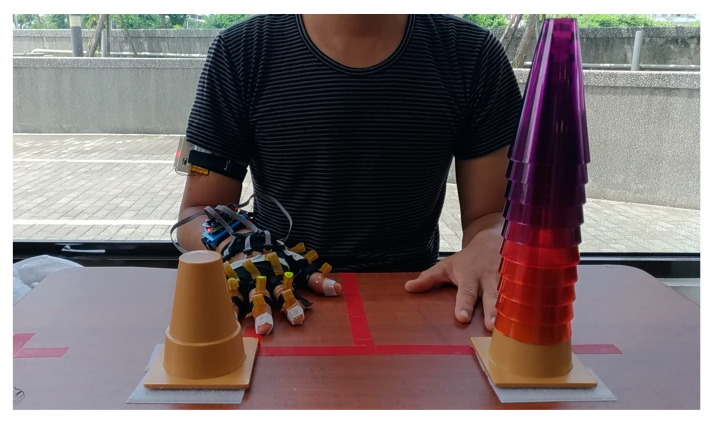
Setup and initial position for the cone-stacking task.

The experimental setup and initial position of the participant for the CS task is presented in Figure 7. Two bases for the cones were placed 20 cm from the edge of the table. The distance between the two bases was 20 cm. Before the task, ten cones were placed on the base on the side opposite to the participant’s test side, that is, if the left side were to be tested, the ten cones were placed on the cone base on the right side. The participants were asked to sit on a chair without a backrest. The chair was adjusted to the height of the participant. On receiving the signal to start the test, the participant moved the cones one by one from one cone base to the other as quickly as possible. The participants were not allowed to lean their trunks to any side during the test. After moving all the cones, the participant returned the test hand to the initial position.

### 3.2. S-FE Task

**Figure 8:**
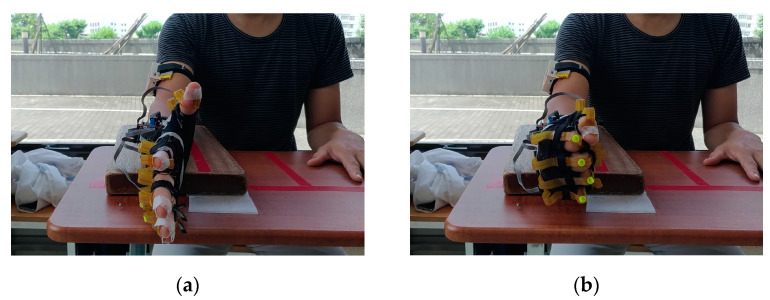
Demonstration of finger flexion and extension: (**a**) full extension and (**b**) full flexion.

The tool for S-FE was a wooden platform of dimensions 36 × 20 × 3 cm^3^. A C-clamp was used to fasten the wooden platform to the table if necessary. The participant placed the test forearm on the wooden platform such that the palm faced inward and the hand was not in contact with the platform or table. The initial position for the task is presented in Figure 8a. The participant had to flex and extend their fingers over their maximum range of motion 50 times at a pace of once per second (Figure 8).

### 3.3. F-FE Task

The setup for the F-FE task was the same as for the S-FE task (Figure 8). The participant placed the test forearm on the wooden platform with the palm in the air. The participant then flexed and extended their fingers 50 times as quickly as possible after hearing the signal to begin the experiment. The participants had to ensure that their fingers reached their maximum joint angles during flexion and extension.

### 3.4. S-BS Task

**Figure 9:**
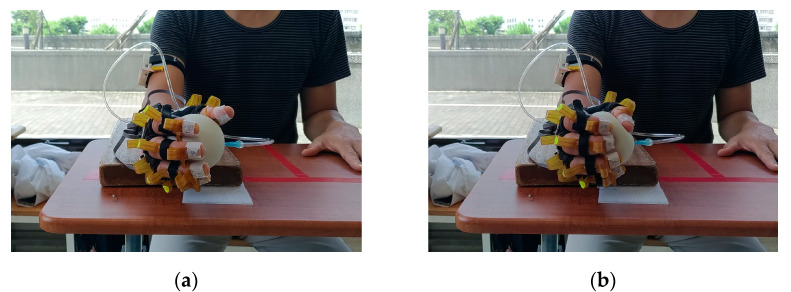
Ball-squeezing tasks: (**a**) initial position and (**b**) squeezing the ball.

The setup for the S-BS task was the same as that for the S-FE and F-FE tasks. The participant placed the forearm of the test side on the wooden platform with the palm hanging freely in the air. The participant held the tennis ball lightly without applying force, as presented in Figure 9a. The participant then squeezed the tennis ball with the maximum force that they could muster 50 times slowly (Figure 9b).

### 3.5. F-BS Task

The setup for the F-BS task was the same as that for the S-BS task. The participant was asked to place their forearm on the wooden platform with the palm free in the air. The participants held the soft tennis ball lightly without applying force (Figure 9a). They then squeezed the ball 50 times as quickly as possible (Figure 9b).

### 3.6. Statistical Analysis

This pilot study aimed to investigate whether the proposed system can assess the severity of spasticity in patient with stroke fingers. Therefore, statistical data for characterizing participant voluntary movement was obtained for analysis. The total magnitude of the acceleration, angular velocity, and magnetic field were calculated because the hand movements are in three dimensions.

After the magnitudes of the raw data were obtained, several time-domain and frequency-domain features based on descriptive statistics were extracted from the dataset and their utility for differentiating the two groups of participants was investigated because descriptive statistics can quantitatively describe the features from a collection of information, which was also adopted in our previous study relating map motion features to energy expenditure [19]. For time-domain features, three types of features were extracted, namely, centrality, variability, and shape. Centrality features were the mean and median of the data series; variability features were the interquartile range (IQR), standard deviation (SD), coefficient of variation (CV), and root-mean-square (RMS); and shape features were skewness and kurtosis. For the frequency-domain features, the fast Fourier transform was applied to the time-series data to obtain the main frequency and amplitude of data in the frequency domain. Twelve features, including the first six main frequencies and their amplitudes, were adopted. For the BS tasks, the aforementioned 20 features were also extracted from the raw data of air pressure.

In total, 1140 features were extracted for CS, S-FE, and F-FE, and 1160 features were extracted for S-BS and F-BS. Because few people participated in the pilot study, the Wilcoxon signed-rank test [20] was adopted to determine the significant features for discriminating between healthy participants and patients with stroke. A feature was significant if *p* < 0.05.

## 4. Results

All participants completed the CS, S-FE, F-FE, S-BS, and F-BS tasks. S6 and S7 could not finish the CS task. S3 and S4 were patients with stroke but without spasticity; thus, S3 and S4 were considered healthy participants in the statistical analysis. 

### 4.1. Significant Features of CS

Table 2 presents the significant features for every IMU during the CS task. For all IMUs, the only significant centrality feature was the mean of the gyroscope data; the significant variability features were the IQR, SD, and RMS of angular velocity; the significant frequency features were the amplitude of all main frequencies of acceleration, which are significant. The sixth main frequency of angular velocity and the amplitude of all main frequencies of angular velocity were significant. This result also reveals that shape features were nonsignificant for differentiating healthy participants from patients with stroke with spasticity.

### 4.2. Significant Features of FE Tasks

Table 3 presents the significant features for all IMUs during the S-FE and F-FE tasks. For S-FE, the significant centrality features were the mean and median of angular velocity for all IMUs; the only significant variability feature was the IQR of angular velocity. The shape and frequency features were nonsignificant for differentiating between the healthy participants and the patients with stroke with spasticity in S-FE.

For the F-FE task, for all IMUs, the only significant centrality feature for all IMUs was the mean of angular velocity; the significant variability features were the SD of acceleration and angular velocity and the RMS of angular velocity. The significant frequency features were the amplitude of all main frequencies of acceleration, the second and third main frequencies of angular velocity, and the amplitudes of the first and third through sixth main frequencies of angular velocity. Shape features were nonsignificant for differentiating between the healthy participants and the patients with stroke with spasticity in this test.

### 4.3. Significant Features of BS Tasks

Table 4 presents the significant features for all IMUs during the S-BS and F-BS tasks. For S-BS, no feature was significant for all IMUs. However, the pressure ball features were significant. The significant variability features were the IQR, SD, CV, and RMS of air pressure. The only significant shape features were the skewness of pressure. The significant frequency features were the first through fourth main frequencies and the amplitudes of all main frequencies.

For the F-BS task, the only significant centrality feature was the median pressure. The significant variability features were the SD and CV of air pressure. The only significant shape feature was the skewness of air pressure. The significant frequency features were the amplitude of the first main frequency of angular velocity, the first through fifth main frequencies, and the amplitudes of all main frequencies of air pressure.

### 4.4. Comparison among Participants with Different Finger Spastic Scores

The Wilcoxon signed-rank test was used to identify significant features for discriminating between healthy participants and patients with stroke with spasticity. However, the final goal of this research is to determine the severity of finger spasticity between patients. Hence, the most significant feature for each task was selected according to the *p* value of the Wilcoxon signed-rank test. The most significant features were defined as the features with the smallest *p* value. The same smallest *p* value was shared among several features; thus, one of the kinematic features was selected as the representative for each task for the comparison. However, it does not mean this feature is the only significant one for the task. The Kruskal–Wallis test was adopted to test whether the selected feature could be used to classify the participants into three levels of spasticity based on MAS score [21]. The average value of the selected feature was also calculated for the participants with each spasticity score to visualize the differences between groups. For the BS tasks, the most significant force features were also used for comparison. The selected features and their *p* value in the two tests for each task are listed in Table 5. The kinematic features selected for CS, S-FE, F-FE, S-BS, and F-BS were the SD of the angular velocity of IMU9 (GyroXYZ_9_SD), IQR of the angular velocity of IMU8 (GyroXYZ_8_IQR), skewness of the acceleration of IMU7 (AccXYZ_7_Skewness), amplitude of the first main frequency of the magnetic field of IMU10 (MagXYZ_10_Amp1), and the first main frequency of the angular velocity of IMU12 (GyroXYZ_12_Mainfreq1). The force feature selected for S-BS and F-BS was the CV of air pressure (Ball_Offset_CV) because it was the most significant feature for both S-BS and F-BS. As indicated in Table 5, the features for differentiating the healthy participants from patients with stroke with spasticity and the features for differentiating spasticity scores all had *p* values under 0.05, indicating that the selected features can be used to classify the participants.

## 5. Discussion

This pilot study introduces a novel wearable system and investigated which kinematic features obtained from the system can be used to assess finger spasticity. The participants were asked to perform five voluntary movements, and the corresponding kinematic and force data were collected. The results in Table 2 and Table 3 reveal that the centrality and variability of angular velocity of all IMUs significantly differed between healthy participants and patients with stroke with spasticity in the CS, S-FE, and F-FE tasks. The participant faces no external resistance in these three tasks. Therefore, the participant can undertake their maximum range of motion, and the data can be further used to assess if movement is affected by spasticity. In particular, the mean of angular velocity was significant for every sensor in the three tasks; thus, it is a key feature for differentiating between healthy participants and patients with spasticity. Moreover, the variability of the IMUs data is also a key characteristic for differentiation. A possible reason is that the patients with stroke with spasticity cannot perform the three tasks quickly due to abnormal muscle contractions; this effect would not be present in healthy participants.

Table 4 indicates that the pressure ball features were more significant than those from the IMUs for the BS tasks. The variability, shape, and frequency features for the pressure ball were all significant for differentiating between healthy participants and patients with spasticity in these tasks. This result is reasonable because the two tasks focused on ball squeezing instead of finger movement quality. Furthermore, the resistance of the pressure ball might affect joint movement, resulting in only features from certain IMUs being significant. The overall results also indicate that S-FE had the fewest significant features for all IMUs. Therefore, only the CS, F-FE, S-BS, and F-BS tasks should be adopted in further studies.

Table 5 presents the selected features and *p* values for differentiating between two or three different spasticity scores. The results reveal that the *p* value for all selected features were less than 0.05, indicating that these features are differentiating ones. The overall results indicate that the data collected by the wearable system has potential for assessments of the spasticity scores in future research.

Table 6 shows the comparison between our proposed system and other related works using IMUs to assess spasticity. Among all the studies, only Ang et al. applied IMUs to assess the lower-limb spasticity, whereas the other studies applied IMUs to assess upper-limb spasticity. Regarding the devices, our system adopted most sensors among all the related studies, indicating that this system can measure and analyze most joints compared to other studies. Besides kinematic data, our system can also measure force data, which is the first study to combine the two aspects of information in one system. Our system also had higher portability and extensibility compared to the other systems, meaning that our system can be combined with more sensors in future works. Moreover, our system and the system proposed by Zhang et al. adopted in our study were customized, which would be cheaper than the business products adopted by the other studies. For the type of transmission, Choi et al., Kim et al. and our system used Bluetooth wireless transmission, which is more flexible than Zhang et al. and Ang et al., who adopted the wired transmission. Moreover, it is also superior to the radio frequency (RF) communication adopted by Chen et al. since RF requires the other platform to receive all the information from the sensors. It is also superior to the 2.4 GHz wireless connection adopted by Ang et al., which also requires a platform to receive the data. For the type of task, we and Chen et al. evaluated the spasticity through voluntary movement, which has higher potential to apply to residual rehabilitation, whereas the other studies assessed spasticity through passive movement, which can only be performed by therapists in the follow-up appointment. Lastly, other studies mainly focused on assessing wide joints, such as shoulder, elbow, wrist, and thumb, whereas our study focused on assessing finger spasticity. In conclusion, this is the first study to develop a wearable system for discovering the relations between finger spasticity and voluntary movement, which was crucial but seldom discussed in the related research.

To the best of our knowledge, the developed system is the first wearable system for assessing finger spasticity. This pilot study had few participants because its purpose was to verify the feasibility of the new wearable system for assessing finger spasticity. For a new customized wearable system, verifying whether the kinematic and force data collected from the wearable system are reliable for assessing the finger spasticity is crucial before future studies are conducted. The features extracted from the data obtained from the wearable system could be used to differentiate between participants with different finger spasticity scores. However, the number of subjects in this study is insufficient for training a machine learning model. Thus, in the future, more subjects’ information will be collected using the developed system for building the machine learning model to assess the spasticity. Furthermore, because more significant features were obtained from the CS, F-FE, S-BS, and F-BS, these four tasks should be adopted to efficiently collect kinematic and force data in future studies.

## 6. Conclusions

This study proposed a novel system that employs multiple sensors to assess finger spasticity in patients with stroke. The results reveal that the significant features extracted from five tasks can be used to discriminate the participants with different finger spastic levels. The results demonstrate a close relationship between spasticity and voluntary movement. In particular, more significant features were obtained from the CS, F-FE, S-BS, and F-BS tasks, indicating that the four tasks can be adopted in further studies for collecting information from more participants. Since the aim of this study was to investigate the feasibility of the proposed system, the number of participants recruited in this study was insufficient for building a model to assess finger spasticity. Therefore, more participants should be recruited in a future study to construct a robust model for MAS score prediction for fingers.

## Figures and Tables

**Table 1:** Demographic information of the participants.

No.	Age	Gender	Dominant Side	Months Since Stroke	Type of Stroke	Affected Side	MAS of Fingers’ MCP
N1	21	Female	Right	None	None	None	0
N2	21	Female	Right	None	None	None	0
N3	21	Female	Right	None	None	None	0
N4	21	Male	Right	None	None	None	0
N5	43	Male	Right	None	None	None	0
N6	50	Female	Right	None	None	None	0
N7	44	Male	Right	None	None	None	0
S1	50	Male	Right	33	Infarct	Right	1
S2	80	Female	Right	12	Infarct	Left	1
S3	58	Female	Right	44	Infarct	Right	0
S4	70	Male	Right	9	Infarct	Right	0
S5	47	Male	Right	11	Hemorrhage	Left	2
S6	66	Female	Right	26	Infarct	Left	2
S7	57	Female	Right	59	Infarct	Right	2

**Table 2:** The significant features to discriminate patients of stroke and healthy subjects for CS task.

Feature Type	Sensor Type	Statistics
Centrality	Gyroscope	Mean
Variability	Gyroscope	IQR
	Gyroscope	SD
	Gyroscope	RMS
Frequency	Accelerometer	Amp1 to 6
	Gyroscope	Mainfreq6
	Gyroscope	Amp1 to 6

**Table 3:** The significant features to discriminate patients of stroke and healthy subjects for FE tasks.

	Feature Type	Sensor Type	Statistics
S-FE	Centrality	Gyroscope	Mean
		Gyroscope	Median
	Variability	Gyroscope	IQR
F-FE	Centrality	Gyroscope	Mean
	Variability	Accelerometer	SD
		Gyroscope	SD
		Gyroscope	RMS
	Frequency	Accelerometer	Amp1 to 6
		Gyroscope	Mainfreq2 to 3
		Gyroscope	Amp1, Amp3 to Amp6

**Table 4:** The significant features to discriminate patients of stroke and healthy subjects for BS tasks.

	Feature Type	Sensor Type	Statistics
S-BS	Variability	Pressure Ball	IQR
			SD
			CV
			RMS
	Shape	Pressure Ball	Skewness
	Frequency	Pressure Ball	Mainfreq1 to 4
			Amp1 to 6
F-BS	Centrality	Pressure Ball	Median
	Variability	Pressure Ball	SD
			CV
	Shape	Pressure Ball	Skewness
	Frequency	Gyroscope	Amp1
		Pressure Ball	Mainfreq1 to 5
		Pressure Ball	Amp1 to 6

**Table 5:** The most significant features for each task.

Task	Selected Feature	*p*-Value For Two Spastic Levels	*p*-Value for Three Spastic Levels
CS	GyroXYZ_9_SD	0.013	0.042
S-FE	GyroXYZ_8_IQR	0.003	0.009
F-FE	AccXYZ_7_Skewness	0.003	0.011
S-BS	MagXYZ_10_Amp1	0.003	0.010
	Ball_Offset_CV	0.003	0.009
F-BS	GyroXYZ_12_Mainfreq1	0.003	0.009
	Ball_Offset_CV	0.003	0.009

**Table 6:** Comparison between our proposed system and other related works.

	Our System	Choi et al. [22]	Ang et al. [9]	Zhang et al. [11]	Kim et al. [12]	Chen et al. [15]
Devices	19 IMUs + 1 Pressure Ball	2 IMUs	3 IMUs + 1 sEMG	1 IMU + 2 sEMG	1 IMU	3 IMUs + 3 sEMG
Portability	High	High	High	Low	High	Low
Extensibility	High	Low	Low	Low	Low	Low
Cost	Low	High	High	Low	High	High
Observed Joints	16	2	4	1	1	1
Collected Data Type	Kinematic + Force	Kinematic	Kinematic	Kinematic + EMG	Kinematic	Kinematic + EMG
Transmission	Bluetooth v2.1+EDR	Class 2 Bluetooth	2.4 GHz Wireless	Wired	Bluetooth v2.1+EDR	Radio Frequency communication
Type of Tasks	Voluntary	Passive	Passive	Passive	Passive	Voluntary
Assessed Joints	Fingers	Knee, Ankle	Shoulder, Elbow, Wrist, Thumb	Elbow	Elbow	Elbow

## Data Availability

Not applicable.

## References

[1] Sommerfeld D.K., Gripenstedt U., Welmer A.K. (2012). Spasticity after stroke: An overview of prevalence, test instruments, and treatments. Am. J. Phys. Med. Rehabil..

[2] Dietz V., Sinkjaer T. (2007). Spastic movement disorder: Impaired reflex function and altered muscle mechanics. Lancet Neurol..

[3] Plantin J., Pennati G.V., Roca P., Baron J.-C., Laurencikas E., Weber K., Godbolt A.K., Borg J., Lindberg P.G. (2019). Quantitative Assessment of Hand Spasticity after Stroke: Imaging Correlates and Impact on Motor Recovery. Front. Neurol..

[4] Bohannon R.W., Smith M.B. (1987). Interrater reliability of a modified Ashworth scale of muscle spasticity. Phys. Ther..

[5] Craven B.C., Morris A.R. (2010). Modified Ashworth scale reliability for measurement of lower extremity spasticity among patients with SCI. Spinal Cord.

[6] Ansari N.N., Naghdi S., Arab T.K., Jalaie S. (2008). The interrater and intrarater reliability of the Modified Ashworth Scale in the assessment of muscle spasticity: Limb and muscle group effect. NeuroRehabilitation.

[7] Meseguer-Henarejos A.B., Sánchez-Meca J., López-Pina J.A., Carles-Hernández R. (2018). Inter- and intra-rater reliability of the Modified Ashworth Scale: A systematic review and meta-analysis. Eur. J. Phys. Rehabil. Med..

[8] Park H.S., Kim J., Damiano D.L. (2012). Development of a haptic elbow spasticity simulator (HESS) for improving accuracy and reliability of clinical assessment of spasticity. IEEE Trans. Neural Syst. Rehabil. Eng..

[9] Ang W.S., Geyer H., Chen I.M., Ang W.T. (2018). Objective Assessment of Spasticity with a Method Based on a Human Upper Limb Model. IEEE Trans. Neural Syst. Rehabil. Eng..

[10] Park J.-H., Kim Y., Lee K.-J., Yoon Y.-S., Kang S.H., Kim H., Park H.-S. (2019). Artificial Neural Network Learns Clinical Assessment of Spasticity in Modified Ashworth Scale. Arch. Phys. Med. Rehabil..

[11] Zhang X., Tang X., Zhu X., Gao X., Chen X., Chen X. (2019). A regression-based framework for quantitative assessment of muscle spasticity using combined emg and inertial data from wearable sensors. Front. Neurosci..

[12] Kim J.-Y., Park G., Lee S.-A., Nam Y. (2020). Analysis of Machine Learning-Based Assessment for Elbow Spasticity Using Inertial Sensors. Sensors.

[13] Koh C.-L., Pan S.-L., Jeng J.-S., Chen B.-B., Wang Y.-H., Hsueh I.-P., Hsieh C.-L. (2015). Predicting recovery of voluntary upper extremity movement in subacute with severe upper extremity paresis. PLoS ONE.

[14] Bai L., Pepper M.G., Yan Y., Phillips M., Sakel M. (2021). Quantitative measurement of upper limb motion pre-and post-treatment with Botulinum Toxin. Measurement.

[15] Chen Y., Yu S., Cai Q., Huang S., Ma K., Zheng H., Xie L. (2021). A Spasticity Assessment Method for Voluntary Movement using Data Fusion and Machine Learning. Biomed. Signal Process. Control.

[16] Lin B.-S., Lee I.-J., Chen J.-L. (2020). Novel Assembled Sensorized Glove Platform for Comprehensive Hand Function Assessment by Using Inertial Sensors and Force Sensing Resistors. IEEE Sens. J..

[17] MPRLS Ported Pressure Sensor Breakout. https://cdn-learn.adafruit.com/downloads/pdf/adafruit-mprls-ported-pressure-sensor-breakout.pdf.

[18] Simple and Effective Magnetometer Calibration. https://github.com/kriswiner/MPU6050/wiki/Simple-and-Effective-Magnetometer-Calibration.

[19] Lin B.-S., Wang L.-Y., Hwang Y.-T., Chiang P.-Y., Chou W.-J. (2019). Depth-Camera-Based System for Estimating Energy Expenditure of Physical Activities in Gyms. IEEE J. Biomed. Health Inform..

[20] Woolson R.F., D’Agostino R.B., Sullivan L., Massaro J. (2007). Wilcoxon Signed-Rank Test. Wiley Encyclopedia of Clinical Trials.

[21] Ostertagová E., Ostertag O., Kováč J. (2014). Methodology and Application of the Kruskal-Wallis Test. Appl. Mech. Mater..

[22] Choi S., Shin Y.B., Kim S.Y., Kim J. (2018). A novel sensor-based assessment of lower limb spasticity in children with cerebral palsy. J. Neuroeng. Rehabil..

